# Peritraumatic Distress Affected the Course of Depressive Symptoms During the COVID-19 Pandemic Lockdown in Subjects with Affective and Anxiety Disorders

**DOI:** 10.3390/ijerph22040593

**Published:** 2025-04-10

**Authors:** Claudia Carmassi, Carlo Antonio Bertelloni, Valerio Dell’Oste, Sara Fantasia, Lucia Maggioni, Mirella Ruggeri, Branko Ristic, Chiara Bonetto, Sarah Tosato

**Affiliations:** 1 Department of Clinical and Experimental Medicine, University of Pisa, 56126 Pisa, Italy; carlo.ab@hotmail.it (C.A.B.); valerio.delloste@gmail.com (V.D.); dr.fantasiasara@gmail.com (S.F.); 2 Section of Psychiatry, Department of Neuroscience, Biomedicine and Movement Sciences, University of Verona, 37124 Verona, Italy; lucia.maggioni@univr.it (L.M.); mirella.ruggeri@univr.it (M.R.); branko.ristic@univr.it (B.R.); chiara.bonetto@univr.it (C.B.); sarah.tosato@univr.it (S.T.)

**Keywords:** depression, peritraumatic distress, COVID-19, affective disorders, mood disorders, anxiety

## Abstract

In western countries, the COVID-19 pandemic represented a unique scenario of a global threat of contagion of a life-threatening illness, as confirmed by the need for exceptional and never adopted measures represented by national lockdowns. This study aimed to investigate peritraumatic distress in the framework of the lockdown and to measure the impact on the course of depressive symptoms during the “first wave” of the COVID-19 pandemic in Italy. A sample of 131 subjects (52.7% females; mean age 47.0 ± 15.9 years), looking for a first or follow-up psychiatric visit at the outpatient psychiatric services of two Italian university hospitals, was recruited between 1 June 2020 and 30 July 2020 and assessed by the Hamilton Rating Scale for Depression (HAM-D) and the Peritraumatic Distress Inventory (PDI) at the time of enrolment in the study (T0). The HAM-D was administered again after 3 months (T1). Higher PDI scores significantly predicted the persistence or worsening of depressive symptoms. These results give further evidence of the possible interplay between peritraumatic distress and depressive symptoms in the framework of a global health threat such as the COVID-19 pandemic.

## 1. Introduction

The literature has highlighted the vulnerability of subjects with mood disorders to stressful or traumatic events [1,2,3]. For example, in patients with a bipolar disorder (BD), a history of traumatic experiences was present in 98% of the cases [4], influencing the clinical presentation and course of the disorder. Negative life events, in fact, are related to a more severe psychopathology, mood episode relapses, and a worse course of the disease [5,6]. On the other hand, the major depressive disorder (MDD) represents one of the most frequent psychiatric sequelae of a traumatic event [2]. Usually, MDD coexists with anxiety disorders (AD) or post-traumatic stress disorder (PTSD). In such cases, MDD usually encompasses feelings of guilt and shame, pessimistic reflections, intense ruminations on the causes and consequences of the trauma, and sometimes a lack of meaning in life [7].

Peritraumatic distress experienced at the time of the trauma was reported as a risk factor for the development of depressive symptoms [8,9]. It represents the physiological, emotional, and cognitive responses occurring in the context of a traumatic event [10]. A relationship between peritraumatic distress and the subsequent depressive symptoms was observed in survivors of vehicle accidents or natural disasters [10]. The term peritraumatic distress is defined as a set of physiological, emotional, and cognitive responses that occur at and immediately after the time of a trauma; despite originally being adopted as a measure of DSM-IV-TR Criterion A2 for PTSD, it assesses a broader range of such reactions, including subjective immediate responses to trauma, particularly when explored through the operationalized construct defined in validated instruments such as the Peritraumatic Distress Inventory (PDI). In this way, peritraumatic distress could be conceptualized as a construct that comprehensively includes an individual’s subjective experience during and immediately after a traumatic experience, and helps in the diagnosis of trauma-related sequelae including, but not limited to, PTSD.

A previous study pointed out that the COVID-19 pandemic, with the threat of contagion of a potentially life-threatening illness, as well as the related containment measures, could have represented a traumatic experience [11,12], and peritraumatic distress related to the pandemic has been found to be associated with mental health symptoms in exposed populations. In an online survey of 525 subjects, a relationship between peritraumatic distress due to the COVID-19 pandemic and the presence of depressive symptoms was found, especially in participants with pre-existing mental health problems [9]. Indeed, individuals with mood or anxiety disorders appeared to be more vulnerable to the psychological effects of the pandemic [13], and it could be plausible that they experienced more distress related to that event. Indeed, relevant symptoms of depression, besides anxiety and PTSD, were found in patients with mood or anxiety disorders in the aftermath of the first national lockdown [14]. However, scant data are available on the effect of peritraumatic distress due to the pandemic on depressive symptoms in clinical populations.

Thus, the major aim of the present study was to investigate the symptoms of peritraumatic distress, related to the experience of the threat of the contagion of the new and unknown COVID-19 disease and risk of death in the early first phase of the pandemic. The present study also aimed to assess clinically significant depressive symptoms in a cohort of subjects with affective and anxiety disorders three months after the first national lockdown in Italy and to explore whether peritraumatic distress assessed in the immediate aftermath of the first wave and national lockdown could be related to the course of depressive symptoms.

## 2. Materials and Methods

We enrolled all subjects with a current DSM-5 diagnosis of bipolar disorder (BD), major depressive disorder (MDD), or anxiety disorder (AD) in the study. Subjects meeting the diagnostic criteria were consecutively enrolled in the study from 1 June 2020 to 30 July 2020, in the immediate aftermath of the so-called “first wave” of the COVID-19 pandemic in Italy and the related national lockdown. Exclusion criteria were age <18 years and intellectual disability. All individuals, seeking a first or follow-up psychiatric visit, were recruited at the outpatient psychiatric services of two Italian university hospitals, located in Pisa (Tuscany region) and Verona (Veneto region). All subjects were assessed at the time of enrolment in the study (T0) and after 3 months (T1). A detailed description of the recruitment strategy and methods are detailed elsewhere [14].

All participants gave written informed consent after receiving a detailed description of the study. The study was conducted in accordance with the Declaration of Helsinki and approved by the Ethics Committee of Area Vasta Nord-Ovest Toscana (n. 17152/2020) and of the Provinces of Verona and Rovigo (n. 26045/2020).

At T0 and T1, all participants were assessed by means of the Hamilton Rating Scale for Depression (HAM-D) [15], the most widely used clinician-administered scale to evaluate the presence and the severity of depressive symptoms. The scale was administered by a senior psychiatrist. It consists of 21 items, and the total score is derived from the first 17 items, with a score over 14 suggesting the presence of clinically significant depressive symptoms.

At T0, participants also fulfilled the Peritraumatic Distress inventory (PDI) [16]. The PDI is a self-report instrument developed to measure the distress experienced by the subject retrospectively, at the time of the potentially traumatizing event, or immediately after. It is composed of 13 items, each scored on a 5-point Likert-type scale, ranging from 0 (not at all) to 4 (extremely true), with the total score ranging from 0 to 52 and higher scores indicating increased distress. The Italian version of the PDI demonstrates good test–retest reliability, convergent and divergent validity and good internal consistency [17]. Moreover, a series of socio-demographic and clinical characteristics (medications, comorbidity, COVID-19 infection) were collected.

Descriptive analyses were obtained by using frequencies and percentages for the categorical variables and means (standard deviations) for continuous variables. Based on the score at the HAM-D in each evaluation point, the sample was divided into two groups: those whose score was over 14 at T1 (including patients with a persistent presence of depressive symptoms and patients who worsened at follow-up) [Depressed group at T1], and those whose score was below or equal to 14 at T1 (including patients with a stable absence of clinically relevant depressive symptoms and patients who improved at follow-up) [Not Depressed group at T1].

The two groups were compared using the Chi-square test for categorical variables and the *t*-test for independent samples for continuous variables. A series of univariate logistic regression models were estimated with the group defined by HAM-D at T1 as the dependent variable and each sociodemographic, clinical, and COVID-19-related characteristic as the independent variable (including PDI score at T0). The independent variables with a *p*-value < 0.05 were entered in a multivariate logistic regression model with the group defined by HAM-D at T1 as the dependent variable. A two-tailed *p*-value test < 0.05 was considered statistically significant. Analyses were performed by Stata 17 for Windows.

## 3. Results

The present study included a sample of 131 subjects (*n* = 69, 52.7% of females) with a current DSM-5 diagnosis of a bipolar disorder (BD; *n* = 85, 64.9%), a major depressive disorder (MDD; *n* = 26, 19.8%) or an anxiety disorder (AD; *n* = 20, 15.3%) (see Table 1). The mean age was 47.0 (15.9) years. More than 40% of the subjects were married, and almost 70% lived with their family. Only 18% of subjects had a degree or a higher educational level. Half the sample was employed (50.4%). The most prevalent diagnosis was a bipolar disorder (64.9%). Most subjects did not experience financial difficulties during the lockdown (84.9%), were not in quarantine or positive for COVID-19 (85.5%), and had no relatives or friends involved with COVID-19 (87.8%). The PDI score at T0 was 13.73 (SD 9.12). The Not Depressed group at T1 was constituted by 82.4% of patients. The only characteristic that significantly differed between the two groups was the PDI score at T0, with the Depressed group showing a higher level of peritraumatic distress (18.09 SD 11.89 vs. 12.81 SD 8.19, *p* = 0.011).

The univariate logistic regression models showed that a higher score of PDI at T0 was the only characteristic correlated with having a HAM-D score above the cutoff at both T0 and T1 or for a clinically significant worsening from T0 to T1 (OR = 1.06, *p* = 0.015; CI95% 1.01–1.11) (data available from the authors).

## 4. Discussion

The main finding of the present study is that higher peritraumatic scores at baseline were related to clinically significant depressive symptoms at both baseline and follow-up or to a clinically significant worsening during the follow-up period. Peritraumatic distress is defined as the emotional, physical and physiological responses experienced during and/or immediately after a traumatic event [16]. Evidence from scientific literature shows how peritraumatic distress is one of the main predictors of the Post-Traumatic Stress Disorder (PTSD) [18], and increasing data suggest how elevated distress among individuals exposed to continuous traumatic experiences is associated with a higher incidence of anxiety, depression and somatization symptoms [19].

The COVID-19 pandemic represented a globally unprecedented condition, which was potentially traumatic due to the increased alarm for the viral spread of an unknown and potentially life-threatening illness and the direct effect of containment measurement (e.g., the lockdown and social restrictions), which led to a constant feeling of threat and perceived loneliness in the general population. This assumption was conceptualized by naming the intensity of people’s immediate physiological, emotional, and cognitive reactions to the pandemic as the COVID-19 peritraumatic distress [20]. A recent study, aiming to evaluate how these distressing events occurring due to COVID-19 impacted mental health, reported a worsening of depressive symptoms in both the general population and people with a pre-existing mental disorder [21]. Given these findings, the results of the present study appear to be in line with the above. In fact, also in the present sample, it was observed how peritraumatic distress due to COVID-19 restrictions is associated with depressive symptoms after 3 months: individuals with clinically significant depressive symptoms at first evaluation were also those who reported a higher traumatic distress. Our evidence corroborates the assumption that the baseline peritraumatic distress levels are associated with the course of depression and anxiety symptoms that have previously emerged in scientific literature [9,22].

When discussing these results, some limitations should be considered. In fact, the relatively low sample size and the lack of an extended multicentric study design could have biased the results and had an implication on the global generalizability of the findings. Other limitations could be represented by the peculiar situation of the COVID-19 pandemic, the short duration of the follow-up, and the lack of assessment of comorbid disorders, as well as the lack of assessments of functional outcome measures. On the other hand, it could certainly be stated as a strength how the present study used validated and reliable assessment tools to characterize both peritraumatic distress and the presence of depressive symptoms.

## 5. Conclusions

In conclusion, despite the limitations mentioned above, the findings of the present study improve our knowledge on the possible role of peritraumatic distress symptoms in psychopathological reactions to potentially severe, stressful situations, particularly for its correlations with depressive symptoms. These results highlight that clinicians must pay attention to the individuals’ acute and persistent peritraumatic reactivity as a response to the COVID-19 pandemic. Circumstances such as the loss of social interactions, constant alertness, and recurrent exposure to traumatic stress, in the framework of a severe risk of contagion for an exceptionally contagious and life-threatening unknown illness, could impair people’s stability in maintaining a safe quality of life and could lead to an increase in mood-related disorders.

## Figures and Tables

**Table 1:** Comparison of sociodemograhic, clinical, and COVID-19 related characteristics between the Not Depressed and the Depressed groups at T1 (*n* = 131).

Characteristics at T0		Total Sample *n* = 131 (100%)	Not Depressed at T1 *n* = 108 (82.4%)	Depressed at T1 *n* = 23 (17.6%)	*p*
Gender	Male	62 (47.3)	52 (48.1)	10 (43.5)	0.819
	Female	69 (52.7)	56 (51.9)	13 (56.5)	
Marital status	Not married	48 (36.6)	39 (36.1)	9 (39.1)	0.928
	Married	56 (42.7)	47 (43.5)	9 (39.1)	
	Divorced/Widowed	27 (20.6)	22 (20.4)	5 (21.7)	
Education (3 missing)	Junior school	50 (39.1)	40 (37.7)	10 (45.5)	0.197
	High school	55 (43.0)	44 (41.5)	11 (50.0)	
	Degree or higher	23 (18.0)	22 (20.8)	1 (4.5)	
Living with family	No	40 (30.5)	33 (30.6)	7 (30.4)	0.991
	Yes	90 (69.5)	75 (69.4)	16 (69.6)	
Employment	Unemployed	30 (22.9)	24 (22.2)	6 (26.1)	0.072
	Employed	66 (50.4)	59 (54.6)	7 (30.4)	
	Other condition	35 (26.7)	25 (23.1)	10 (43.5)	
Psychiatric diagnosis	BD	85 (64.9)	67 (62.0)	18 (78.3)	0.271
	MDD	26 (19.8)	24 (22.2)	2 (8.7)	
	AD	20 (15.3)	17 (15.7)	3 (13.0)	
Financial difficulties due to lockdown (5 missing)	Yes	19 (15.1)	14 (13.2)	5 (25.0)	0.176
	No	107 (84.9)	92 (86.9)	15 (75.0)	
Having been in quarantine or COVID-19-positive	Yes	19 (14.5)	16 (14.8)	3 (13.0)	0.827
	No	112 (85.5)	92 (85.2)	20 (87.0)	
At least one relative/close friend with a COVID-19-related condition	No	115 (87.8)	96 (88.9)	19 (82.6)	0.663
	Infected	5 (3.8)	4 (3.7)	1 (4.3)	
	Deceased	11 (8.4)	8 (7.4)	3 (13.0)	
		Mean (SD)	Mean (SD)	Mean (SD)	
Age (3 missing)		47.0 (15.9)	46.9 (15.9)	47.7 (16.5)	0.824
PDI * score		13.73 (9.12)	12.81 (8.19)	18.09 (11.89)	0.011

* PDI, peritraumatic distress inventory.

## Data Availability

The data that support the findings of this study are available from the corresponding author upon reasonable request.
